# Mutational analysis differentiating sporadic carcinomas from colitis-associated colorectal carcinomas

**DOI:** 10.1186/s12964-024-01856-8

**Published:** 2024-10-10

**Authors:** Theresa Dregelies, Franziska Haumaier, William Sterlacci, Steffen Backert, Michael Vieth

**Affiliations:** 1 Institut für Pathologie, Friedrich-Alexander-Universität Erlangen-Nürnberg, Klinikum Bayreuth, Preuschwitzer Str. 101, 95445 Bayreuth, Germany; 2 Lehrstuhl für Mikrobiologie, Friedrich-Alexander-Universität Erlangen-Nürnberg, Staudtstr. 5, 91058 Erlangen, Germany; 3 Bavarian Cancer Research Center (BZKF), Erlangen, Germany

**Keywords:** Colitis-associated carcinoma, Sporadic carcinoma, Ulcerative colitis, Mutation, Conventional carcinoma, Non-conventional carcinoma

## Abstract

**Background:**

Ulcerative colitis (UC) is a chronic inflammatory bowel disease (IBD) that is associated with increased risk of developing colitis-associated carcinoma (CAC). The genetic profile of CACs is fairly similar to the sporadic colorectal carcinomas (sCRCs), although showing certain differences in the timing and sequence of alterations that contribute to carcinogenesis. Also, both cancer types typically show a strong histological resemblance, which complicates the pathologists’ diagnosis. Due to the different clinical consequences, it is of utmost importance to categorize the corresponding cancer type correctly.

**Methods:**

In this study, we determined the mutation profiles of 64 CACs and sCRCs in the hotspot regions of 50 cancer-associated genes and compared them to 29 controls to identify genetic gene variants that can facilitate the pathologists’ diagnosis. Pearson Chi-Square or Fisher’s exact tests were used for statistical analyses.

**Results:**

We found that sCRCs tend to mutate more frequently in APC and PIK3CA genes than CACs and that mainly males were affected. Our CAC cohort identified the KRAS G12D mutation as group-specific variant that was not detected in the sCRCs. When separating conventional from non-conventional CACs, it was discovered that the conventional type shows significantly more mutations for ATM.

**Conclusions:**

Taken together, our data highlights genetic differences between sCRC and CAC and enables the possibility to utilize specific gene alterations to support the pathologist’s diagnosis.

**Supplementary Information:**

The online version contains supplementary material available at 10.1186/s12964-024-01856-8.

## Introduction

Ulcerative colitis (UC) and Crohn’s disease (CD) represent two forms of inflammatory bowel disease (IBD). UC shows a characteristic inflammatory process that tends to start in the rectum and may ascend continuously into the colon or even the terminal ileum (backwash ileitis). Clinical symptoms include diarrhoea, rectal bleeding and weight loss [1]. With a worldwide prevalence of 84.3 and a death rate of 0.51 per 100,000 people, UC has evolved to a global burden with an increasing rate [2]. So far, the underlying mechanisms of this disease remain widely unclear. It is believed that diet, as well as lifestyle and genetic predisposition, may be involved in the pathogenesis [3]. In the last decade, the question has arisen, whether the microbiota, especially *Fusobacterium nucleatum* (*F. nucleatum*), might be a factor contributing to intestinal cancer development [4, 5].

UC is associated with an increased risk of developing colorectal cancer (CRC). A meta-analysis has shown a relationship between UC and the development of CRC that is based on the duration of the disease. After 10, 20 and 30 years suffering from UC, the risk of cancer development was calculated to be 2%, 8% and 18%, respectively [6]. According to a recent population-based study from Australia, it has been demonstrated that the risk of cancer decreased to 1% after 10 years, 3% after 20 years and 7% after 30 years [7]. In the context of rising incidences of UC, a decrease in the cancer risk might be explained by a more effective therapy and closer follow-up of patients that suffer from UC [8].

The diagnosis in this context is made on results from histology, endoscopy, and clinical symptoms [9]. Based on histology only, it remains difficult to distinguish between CRC that is due to the UC itself, so-called colitis-associated carcinoma (CAC) and sporadic CRC (sCRC) that is a direct cause of somatic mutations, which also applies to the corresponding precancerous lesions [10]. Despite the strong histopathological resemblance of CAC and sCRC, the involved factors of carcinogenesis differ from each other. Due to the inflamed mucosa of the colon, the carcinogenesis of CAC follows an inflammation-dysplasia-carcinoma sequence [11]. The process is driven forward by the formation of dysplasia. Low-grade dysplasia (LGD) and high-grade dysplasia (HGD) represent the precancerous stages of CAC [12]. In contrast to this, sCRC mostly evolves according to the adenoma-carcinoma-sequence, which explains the transformation of corresponding adenoma to carcinoma by the involvement of specific genetic and epigenetic factors [13]. Interestingly, it has been demonstrated that both types of cancer involve similar molecular factors, however, the sequence of arising gene mutations differs widely [12]. A loss-of-function mutation in the tumor suppressor gene *adenomatous polyposis coli* (*APC*) is an initial event in the carcinogenesis of sCRC, while mutations in *KRAS* and *TP53* genes contribute at later stages [13, 14]. In contrast, *APC* mutations represent a rare event in CAC and occur at later stages of carcinogenesis, whereas *KRAS* and *TP53* mutate during early stages [12]. Other genes such as the tumor suppressor F-Box and WD Repeat Domain Containing 7 (FBXW7), the oncogenes Phosphatidylinositol-4,5-Bisphosphate 3-Kinase Catalytic Subunit Alpha (PIK3CA) and to a lesser extent GNAS Complex Locus (GNAS) may also play a role.

However, the histological differentiation between CAC and sCRC is of utmost importance since it has a significant impact on the patient’s treatment scheme. Most cases of sporadic early lesions can be removed endoscopically, although sometimes a surgical resection is considered. In the presence of CAC or precancerous lesions, proctocolectomy is recommended if endoscopic removal is not an option [1]. This greatly affects the patient’s quality of life.

Due to the enormous gaps of our common understanding the carcinogenesis of CAC and the importance of differentiating CAC from sCRC, the need of available biomarkers is irrefutable. Therefore, the present study was designed to identify unique mutational patterns that may support the pathologist’s diagnosis. This mutational dataset was also correlated with a previous study regarding the detection of *F. nucleatum* in this scenario.

## Materials and methods

### Patients and tissue collection

For our study, we included 96 patients that underwent endoscopy or surgery at the Klinikum Bayreuth (Germany) between 2006 and 2020. Biopsies and resection material were assessed at the Institute of Pathology of Klinikum Bayreuth. All samples were collected in a consecutive manner, regardless of tumor size, grade, location or the patients’ age or sex. According to common guidelines and histopathological criteria, tissue samples were diagnosed as UC and UC-associated, as well as sporadic cancerous lesions in the colon (CAC, sCRC) [1, 15]. Diagnoses were evaluated by two independent pathologists, which resulted in consistent conclusions. The study was approved by the ethics committee of the University Bayreuth (#O 1305/1 - GB).

All tissue samples (*n* = 96) were stored in 4% neutral pH-buffered formalin. For preparation of hematoxylin-eosin (HE) staining, biopsies were dehydrated and paraffinized using a HistoCore PELORIS 3 (Leica Biosystems, Germany), followed by microdissection of Formalin-fixed paraffin-embedded (FFPE) blocks to 4 μm. Staining was performed on a Tissue-Tek Prisma apparatus (Sakura Finetek, Japan). According to the histopathological diagnosis, the patients were categorized in four groups: UC (*n* = 17), CAC (*n* = 38), healthy colon (*n* = 15), and sCRC (*n* = 26).

### DNA extraction

Prior to DNA isolation, HE-stained sections of all 96 samples were used to identify regions of interest. To enrich the number of desired cells, FFPE blocks were microdissected to 5 μm and unstained samples were used to extract tissue using disposable scalpels (FEATHER, No. 10). Tissue sections were deparaffinized by incubation for 30 min at 60 °C, followed by washing steps for 20 min each in 97% Xylol (SAV Liquid Production GmbH, Germany) and 96% Ethanol (SAV Liquid Production GmbH, Germany), respectively.

Genomic DNA was isolated using the Maxwell LEV Blood DNA Kit (Promega), carried out on a Maxwell 16 instrument following the manufacturer’s protocol (Promega, Germany). After extraction, the DNA was quantified with the QuantiFluor dsDNA System Kit (Promega, Germany) on a Quantus Fluorometer (Promega, Germany). All DNA samples were stored at -20 °C.

### Next-generation sequencing (NGS)

For library generation, the AmpliSeq for Illumina Cancer HotSpot Panel v2 was used according to manufacturer’s instructions (Illumina, USA). Ten ng of genomic DNA was used per sample for the first PCR. Paired-end sequencing (2 × 150 bp) was carried out on a MiSeq system (Illumina). For detection of somatic gene variants, the sequenced reads of all 96 samples were aligned to the human reference genome GRCh37 (hg19) with the DNA Amplicon app version 2.1.0 using the Burrows-Wheeler Aligner [16]. Afterwards, files were analysed with the Variant Interpreter version 2.6.1.239, using Pisces version 5.2.9.23 for variant identification of non-synonymous somatic mutations such as single-nucleotide polymorphisms (SNPs) and indels (missense, nonsense, frameshift, in-frame coding indels, and splice sites), as well as BaseSpace Annotation Engine version 1.6.2.0 for the annotation (Illumina, USA). Only samples presenting > 100,000 reads and showing variants with a quality score > 90 and a population frequency < 1% were evaluated. Three samples in the UC group did not meet these criteria and were excluded from further investigations.

### Detection of *F. nucleatum*

Ninety samples of the above described patients (CAC: *n* = 35, sCRC: *n* = 26, UC: *n* = 14, and healthy controls: *n* = 15) were tested for the presence of *F. nucleatum* by conducting quantitative real-time polymerase chain reaction (qPCR) as described earlier [17]. This data was further correlated with the mutational status of the corresponding patients.

### Statistical analysis

All statistical analyses were performed with the SPSS tool (IBM SPSS Inc., USA, Version 23.0). Pairwise comparisons were conducted with Pearson Chi-Square test and Fisher’s exact test was used for sample sizes < 5. Exact two-sided tests were performed and *p* values < 0.05 were considered statistically significant. For correlation analysis the contingency coefficient was calculated.

## Results

### Clinicopathological features

**Fig. 1:**
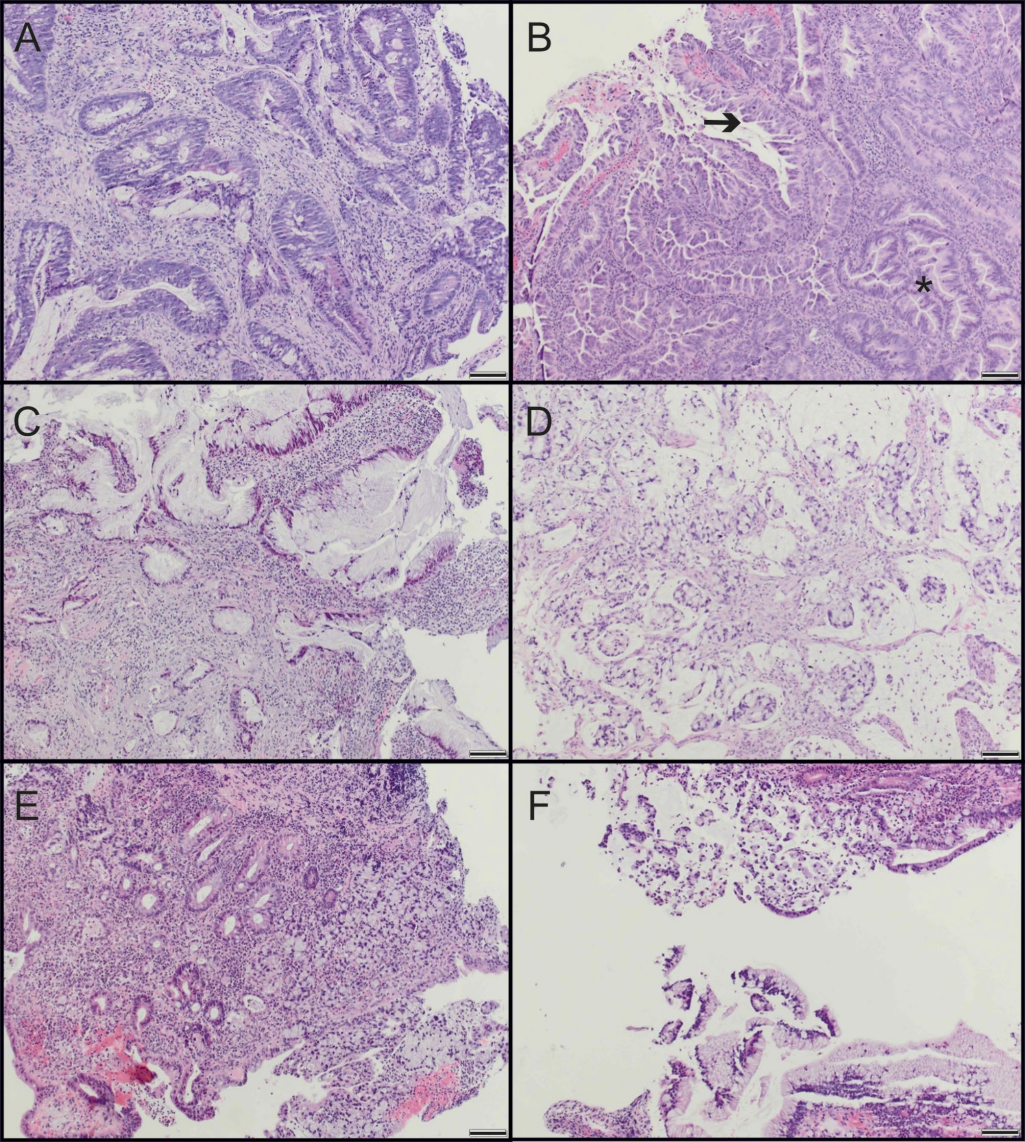
HE-stained images highlight differences between sCRC and CAC. HE images of sporadic conventional (**A**) and ulcerative colitis-associated, non-conventional (**B**-**F**) carcinomas. **B**) Carcinoma with micropapillary tufts (arrow) and serration (asterisk). **C**) Hypermucinous carcinoma. **D**) Mucinous carcinoma with intra- and extracellular mucin. **E**) Signet ring cell carcinoma. **F**) Mixed types: hypermucinous (bottom half) and signet ring cells (top half). Scale bars are equivalent to 100 μm

The histological differentiation between CAC and sCRC is complicated by the fact that both tumors can show similar characteristics. Although colitis-associated dysplasia is often of the sporadic, conventional type, and other distinctive types, non-conventional morphologies are being recognized in patients with IBD (Fig. 1). The most commonly described non-conventional types include hypermucinous, signet ring cell, serrated or micropapillary histology, as well as mixed lesions [18, 19]. Our study included 93 patients of which 48.4% were females and 51.6% were males. The patients showed a median age of 58 years, ranging from 18 to 91 years. All clinicopathological characteristics of the patients are displayed in Table 1.

**Table 1:** Correlation of *F. nucleatum* data with mutation status of most common mutated genes for both cancer groups

	UC (n=14)	CAC (n=38)	Healthy colon (n=15)	sCRC (n=26)
**Sex**				
Male	9 (64.3%)	24 (63.2%)	7 (46.7%)	8 (30.8%)
Female	5 (35.7%)	14 (36.8%)	8 (53.3%)	18 (69.2%)
**Age**				
< 60 years old	9 (64.3%)	20 (52.6%)	10 (66.7%)	7 (26.9%)
≥ 60 years old	5 (35.7%)	18 (47.4%)	5 (33.3%)	19 (73.1%)
**Tumor Localisation**				
Right-sided		8 (21.1%)		12 (46.2%)
Left-sided		30 (78.9%)		14 (53.8%)

In our cohort, CACs were mainly localized in the left-sided colon (78.9%), whereas for sCRCs this was the case in about half of the cases (53.8%). We also observed significant differences in the patients’ age at the initial time of cancer diagnosis. A sCRC seems to develop more likely after the age of 60, which cannot be confirmed for CAC (*p* = 0.041).

### Group-specific mutations were identified for CAC and sCRC

NGS of the hotspot regions of 50 cancer-associated genes was performed on 93 samples, to identify distinct molecular patterns that facilitate the pathologist’s diagnosis. Therefore, the detected gene variants were compared within all four patient groups to determine unique mutations in CAC and sCRC. In total, we identified four and five mutations in the groups of CAC and sCRC, respectively, which were detected in at least two samples (Table 2). Most of these variants occurred in p53 and KRAS, but also MET, APC, and ATM genes, while PIK3CA shows distinct patterns. Interestingly, KRAS G12D was present in 13.2% of CACs, while another amino acid exchange at the same codon, G12A, was detected in 11.5% of sCRCs. In all sCRCs, the group-specific mutation with the highest occurrence of 15.4% was found in APC, resulting in the nonsense variant R1450Ter. Also, p53 tends to mutate an arginine at position 248 whereas the variant R248Q only appeared in the group of CACs, in the sCRC group R248W was identified as a unique mutation. The mutation in MET T1010I was found in 5.3% of CACs, while ATM P604S and PIK3CA Q546K were both detected in 7.7% of analyzed sporadic cancers.

**Table 2:** Unique gene variants identified in at least two samples of CAC and sCRC

CAC				
Mutation				
Gene	Exon	DNA	Protein	Relative Frequency [%]
KRAS	2	c.35G> A	p.G12D	13.2
p53	4	c.216dup	p.V73RfsTer76	5.3
p53	7	c.743G> A	p.R248Q	5.3
MET	14	c.3029 C> T	p.T1010I	5.3
**sCRC**				
APC	16	c.4348 C> T	p.R1450Ter	15.4
KRAS	2	c.35G> C	p.G12A	11.5
p53	7	c.844 C> T	p.R248W	11.5
ATM	12	c.1810 C> T	p.P604S	7.7
PIK3CA	10	c.1636 C> A	p.Q546K	7.7

Amongst all variants, the FBXW7 gene in-frame mutation I355del was detected in most samples. This deletion accounts for 28.0% of all cases and occurred in every sample group. In total, twelve CACs, seven sCRCs, five UCs and two healthy samples revealed this variant. Since healthy colon biopsies also presented this altered FBXW7 protein, this variant was considered as a polymorphism and was excluded from further mutational analysis.

### CAC and sCRC show different mutational patterns

**Fig. 2:**
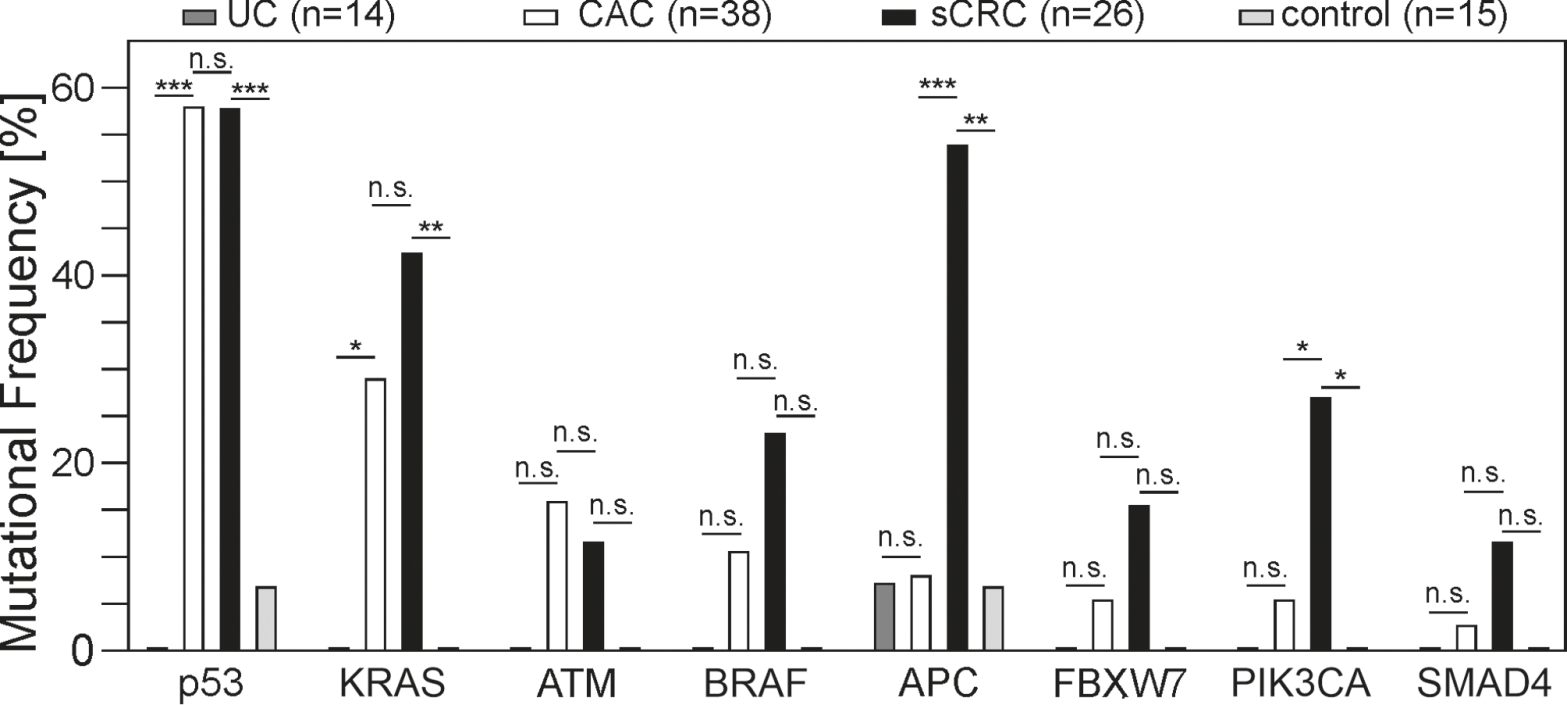
Most frequent mutant genes in all groups. The X-axis displays commonly mutated genes, and the Y-axis indicates the corresponding mutational frequency. Mutational analysis was performed on 93 samples. Within the CAC and sCRC group, p53 showed the highest mutation rates of all analyzed genes, with 57.9% and 57.7%, respectively. APC mutations were detected at a significantly higher rate in sCRC (53.8%) than in CAC (7.9%), which was also observed for PIK3CA. For all other genes, no statistical significance was observed between both cancer types. ***p ≤ 0.001, **p ≤ 0.01, *p ≤ 0.05, n.s. not significant

We further investigated the general frequency of mutated genes and compared this data between both cancer groups and the corresponding control groups (Fig. 2). The most frequent mutations in cancer samples occurred in p53, KRAS, ATM, BRAF, APC, FBXW7, PIK3CA and SMAD4. Less frequently observed mutations occurred in MET, PDGFRA, GNAS, PTEN and CDKN2A.

In general, 85.9% of all 64 cancer samples show at least one mutation in the eight most mutated genes. APC mutation was detected in 53.8% of sCRCs, whereas this was the case in only 7.9% of CACs, which was statistically significant (*p* = 0.000084). Furthermore, sCRCs have a higher tendency to mutate in PIK3CA (26.9%) compared to CACs (5.3%; *p* = 0.025). Interestingly, both types of cancer present the same mutational rate for p53, as 57.9% of CACs and 57.7% of sCRCs were altered (*p* = 0.987). This resulted in the most mutated gene within all sample groups. Next to p53, CACs (15.8%) only presented a higher mutation frequency than sCRCs (11.5%) in ATM (*p* = 0.728). Generally, within the most mutated genes, sCRC samples show a tendency to mutate more frequently than CAC samples, although this observation did not result in statistical significance. In general, mutations in the control and UC group occurred very rarely. APC alterations were detected in 6.7% and 7.1% of healthy control and UC samples, respectively. Also, in one sample of healthy colon biopsy, a p53 missense mutation was detected.

**Fig. 3:**
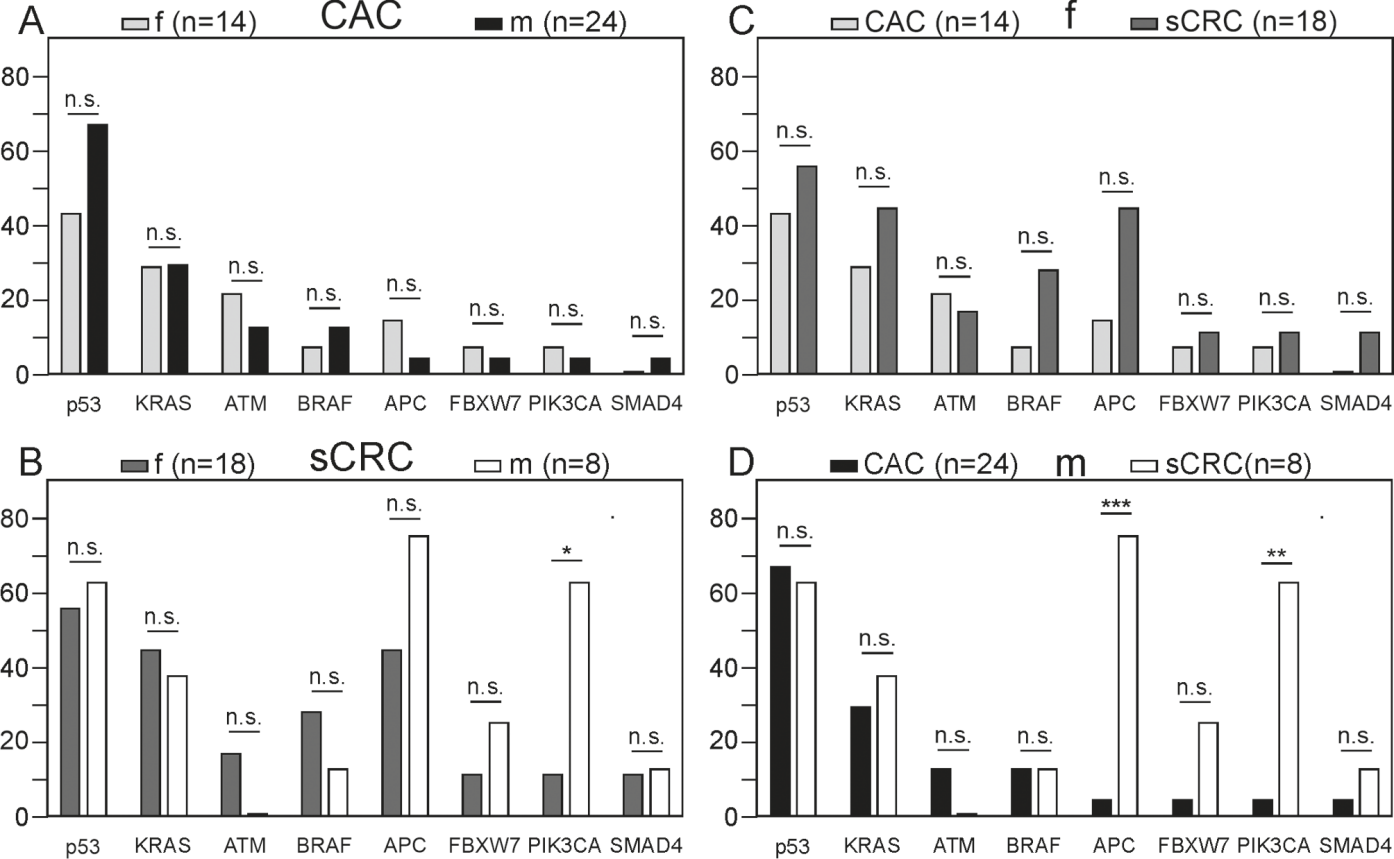
Most frequent mutant genes according to the sex. The X-axis displays commonly mutated genes, and the Y-axis indicates the corresponding mutational frequency. Mutational analysis was performed on 93 samples. The distribution of mutated genes within the sex is displayed for CAC (**A**) and sCRC (**B**). The data is also presented as sexwise comparison within the two cancer groups for females (**C**) and males (**D**). ***p ≤ 0.001, **p ≤ 0.01, *p ≤ 0.05, n.s. not significant

In addition, we investigated whether sex plays a role regarding mutations in certain genes (Fig. 3). For this purpose, the datasets were re-evaluated. We generally observed a slightly higher mutation rate for male patients than for female patients with CRC. In total, 81.3% of female cancer samples and 90.6% of male cancer samples showed at least one mutation in the eight most common mutated genes (*p* = 0.474). Within the CAC group, 87.5% of male and 71.4% of female samples presented gene mutations (*p* = 0.387) (Fig. 3A). The highest observed difference occurred in p53, as 42.9% of females and 66.7% of males showed mutations (*p* = 0.187). No statistically significant differences were detected between females and males of the CAC group. Comparable results were obtained for the second cancer group, the sCRCs, with one significant difference (Fig. 3B). Males that suffer from sCRC tend to have a higher risk to develop mutations in PIK3CA than females. Where 62.5% of male biopsies presented alterations herein, mutations were only present in 11.1% of female samples (*p* = 0.014). Also, 75.0% of males showed a higher mutation rate for APC than females, who were altered in 44.4% of cases (*p* = 0.216). Overall, a low correlation was established between sex and mutation frequency in both cancer groups (CAC: *r* = 0.196, *p* = 0.218; sCRC: *r* = 0.189, *p* = 0.326). A limitation that should be mentioned is the somewhat unbalanced patient cohort, especially in the two CRC groups. It would be helpful to test more female patients with CAC and more male samples with sCRC to verify the observed trends in gene mutations.

Next, we directly compared the mutational status of females and males between sCRC and CAC (Fig. 3C, D). In contrast to male and female patients with CAC, the corresponding samples of sCRC patients tend to present more mutations (females: *p* = 0.365, males: *p* = 0.555). The most important differences were detected for APC and PIK3CA status in male samples. Whereas only 4.2% of male CAC patients were altered in APC and PIK3CA, males with sCRC revealed mutations in 75.0% and 62.5%, respectively (*p* = 0.000202, *p* = 0.002). The significance in mutational status of APC and PIK3CA between the two different cancer types is dominated by male patients with sCRC. For none of the other genes significant values were obtained.

### Mutational profile of KRAS differs between CAC and sCRC

**Fig. 4:**
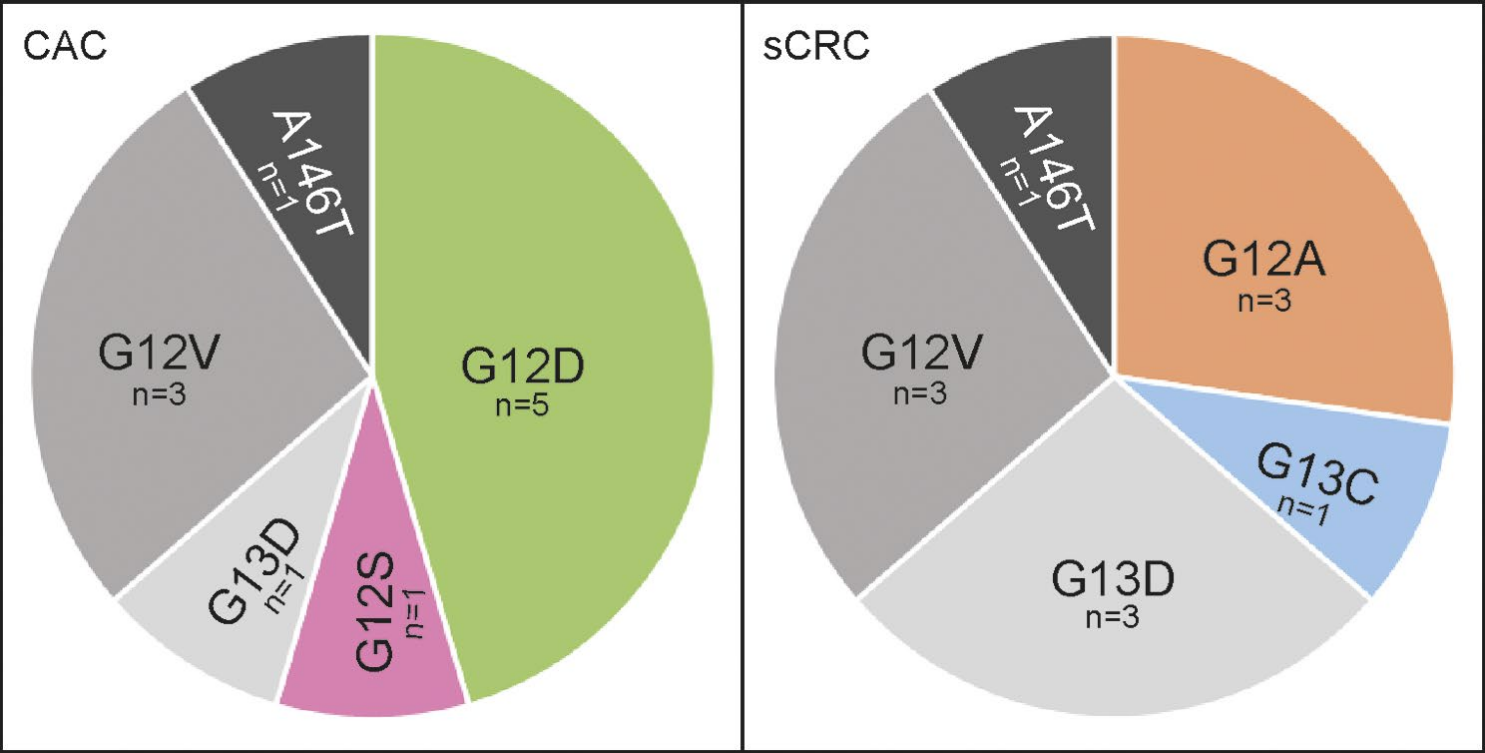
Detected KRAS mutations in CAC and sCRC. In total, 64 CRC samples were analyzed for KRAS alterations. In eleven patient samples from each group a KRAS variation was present. Each cancer group showed two unique mutations that only appeared in one of the two cancer groups. For CAC the unique mutations were G12D (45.5%) and G12S (9.1%), while in sCRC G12A (27.3%) and G13C (9.1%) were detected. The variant G12V was detected in 27.3% in both cancer groups, and A146T in 9.1%

Analysis of the KRAS mutation status revealed differences between CAC and sCRC (Fig. 4). In 28.9% of CAC and 42.3% of sCRC samples a KRAS alteration was present (*p* = 0.269). For the identification of potential gene variants that support the pathologist’s diagnosis, all KRAS variants were screened for group-specific alterations. KRAS mutations found exclusively in CAC were G12D (45.5%) and G12S (9.1%), whereas G12A (27.3%) and G13C (9.1%) were uniquely found in sCRC. G12V (27.3%) and A146T (9.1%) were detected at the same rates in CAC and sCRC. The variant G13D was detected in both sample groups but showed a higher prevalence in sCRC (27.3%) compared to CAC (9.1%) (*p* = 0.586). Interestingly, 81.8% of all KRAS mutations in CAC and 54.5% in sCRC affected codon 12, adding up to 68.2% in both cancer groups. Codon 13 was altered in 22.7% of all cancer samples, presenting the second most frequently altered codon. Of all gene variations, G12D is the only mutation that exhibits statistical relevance between CAC and sCRC (*p* = 0.035).

### The typing of CACs has an impact on the mutation frequencies

CACs can be categorized into conventional and non-conventional carcinomas (Fig. 1). In the next step we investigated whether the CAC types show differences in their mutation status. Our CAC cohort incorporated 38 samples, of which 17 were non-conventional types and 21 corresponded to the conventional type (Table 3). In total, 90.5% of conventional and 88.2% of non-conventional CACs showed at least one mutation of the eight most commonly mutated genes (*p* = 1.000). In particular, non-conventional CACs present a higher mutation rate in p53, BRAF, APC, FBXW7, PIK3CA and SMAD4, whereas conventional tumors show more KRAS and ATM mutations. Interestingly, only conventional CACs showed mutated protein variants of ATM (28.6%), while none of the non-conventional samples was affected by mutations in the corresponding gene (*p* = 0.024). Also, the detection of the KRAS status resulted in clear differences. 42.9% of conventional CACs were affected by KRAS mutations, whereas this was only the case for 11.8% of non-conventional samples (*p* = 0.070). In contrast, non-conventional CACs presented a p53 mutation in 70.6% and conventional tumors in only 47.6% (*p* = 0.197). FBXW7 and SMAD4 mutations were also only found in non-conventional CACs (11.8%; 5.9%), but not in conventional tumors (*p* = 0.193; *p* = 0.447).

**Table 3:** Mutation status of conventional and non-conventional CACs

CAC type	Mutation status (mut/ wt)	Related to all genes	p53	KRAS	ATM	BRAF	APC	FBXW7	PIK3CA	SMAD4
**Conventional**	mut	19	10	9	6	1	1	0	1	0
	wt	2	11	12	15	20	20	21	20	20
	**Total**	**21**								
**Non-conventional**	mut	15	12	2	0	3	2	2	1	1
	wt	2	5	15	17	14	15	15	16	16
	**Total**	**17**								

*mut: mutated, wt: wildtype

### The presence of *F. nucleatum* does not correlate with the patient’s mutational status

In a previous study, we investigated whether the occurrence of *F. nucleatum* is related to the carcinogenesis of CAC or might even serve as a biomarker for the differentiation between CAC and sCRC [17]. In this paper, we additionally analyzed the relationship between the presence of the bacterium and the mutation frequency of certain host genes (Table 4). In total, 14 patients with CAC or sCRC were positive for *F. nucleatum*, whereas 21 and 12 were negative, respectively. In the group of CACs, more patients that were negative for *F. nucleatum* showed protein alterations than samples that were positive for the bacterium. Still only a negligible correlation was detected between the presence of this bacterium and the gene mutation rates (*r* = 0.248, *p* = 0.143). 90.5% of patients within the CAC group that were not affected by *F. nucleatum* were mutated in host tissue, whereas positive samples only showed mutations in 71.4% of cases (*p* = 0.191). For patients that harbor a sCRC, the mutation frequency seems independent of the *F. nucleatum* status, which was also confirmed by correlation analysis (*r* = 0.022, *p* = 0.910). In the sCRC group 92.9% of positive and 91.7% of negative samples were altered in the most mutated genes (*p* = 0.720). By taking a closer look at the individual genes, significant differences were only observed for APC. While none of the *F. nucleatum-*positive CAC samples were mutated, 42.9% of positive sCRC samples were altered in APC (*p* = 0.016). *F. nucleatum*-negative samples for CAC and sCRC were mutated in 14.3% and 66.7%, respectively (*p* = 0.005). This suggests that the *F. nucleatum* status neither has an impact on the mutation rate for APC, nor on other genes analyzed here. Also, our data showed that there is no significant correlation between the sex and the presence of *F. nucleatum* in both cancer groups (CAC: *r* = 0.024, *p* = 0.886; sCRC: *r* = 0.214, *p* = 0.265) [17]. An overview of the whole study design is presented in Fig. S1.

**Table 4:** Correlation of *F. nucleatum* data with mutation status of most common mutated genes for both cancer groups

	F. nucleatum (pos/neg)/ mutation status (mut/ wt)	Related to all genes	p53	KRAS	ATM	BRAF	APC	FBXW7	PIK3CA	SMAD4
**CAC**	Pos/ mut	10	7	4	3	3	0	0	2	1
	Pos/ wt	4	7	10	11	11	14	14	12	13
	Neg/ mut	19	13	7	3	1	3	1	0	0
	Neg/ wt	2	8	14	18	20	18	20	21	21
	**Total**	**35**								
**sCRC**	Pos/ mut	13	9	5	1	5	6	2	5	3
	Pos/ wt	1	5	9	13	9	8	12	9	11
	Neg/ mut	11	6	6	2	1	8	2	2	0
	Neg/ wt	1	6	6	10	11	4	10	10	12
	**Total**	**26**								

*pos: positive, neg: negative, mut: mutated, wt: wildtype

## Discussion

UC is associated with an increased risk for CRC, however, the underlying mechanisms remain widely unknown [11]. Such a carcinoma can either be associated with the UC or might have developed sporadically. Both types of carcinomas exhibit a strong histological resemblance, which complicates the pathologists’ diagnosis. Further medical treatment depends on the diagnosis, which can include endoscopic surgery only, but also might require a proctocolectomy [1]. This highlights the need of molecular biomarkers that assist the pathologists’ diagnosis and subsequent treatment. In this study, we performed NGS on a cohort of 93 samples to reveal differences in the mutational patterns.

While CACs seem to develop according to the “inflammation-dysplasia-carcinoma” sequence [11], sCRCs mostly arise from adenomas [20]. Interestingly, both cancer types share some similarities concerning the involved genes. Differences mainly concern the time-point of mutations, which highlights the different tumor entities. We detected four and five gene variants in the CAC and sCRC groups, respectively, which uniquely appeared in the corresponding group in at least two samples. With a detection rate of 15.4%, the most common unique mutation in the sCRC group occurred in APC and represents the nonsense mutation R1450Ter. The affected codon is located in the mutation cluster region (MCR) that spans from codon 1285 to 1580 [21]. To accompany the pathological diagnosis, we would recommend not to look for a single mutation, but a group of gene variants. By screening for the four most common unique mutations of all CACs, 28.9% would have been assigned correctly to this group. Instead of looking for only one common mutation in the sCRC group, it is possible to rise the number of correctly identified sCRCs from 15.4 to 38.5% or even 53.8% by screening for the three or five most frequent mutations of this group. According to the seven or nine unique mutations with the highest detection rate of both carcinoma groups, 32.8% or 39.1% of cases in the whole cohort, would have been diagnosed correctly as CAC or sCRC. According to these results, we recommend to perform NGS with a panel that covers ATM, APC, KRAS, MET, PIK3CA and p53.

Overall, CAC and sCRC tend to mutate in similar genes, but may differ in the corresponding mutation frequencies. One major role in sCRC formation is the constitutive activation of the WNT/β-catenin signaling pathway, which is initiated by loss-of-function mutations in *apc* [11]. It was reported that sCRCs show a higher mutation rate for this gene compared to CACs [11, 22–24]. Our data is consistent with this statement, as 53.8% of sCRCs and only 7.9% of CACs were mutated for this gene. Another study detected similar, but noticeably higher values for both groups, with 73.4% and 22.2%, respectively [25]. This leads to the assumption that *APC* mutations are dispensable for tumor initiation. Next to this, also variants in *KRAS* seem to be more seldom in CACs compared to sCRCs [11, 22, 23]. A recent study detected a mutation rate of 22.2% and 48.6%, respectively, which showed that *KRAS* mutations occur about twice as often in sCRCs [25]. In our dataset, there was no significant difference in both cancer groups, as 28.9% of CACs and 42.3% of sCRCs were mutated. Interestingly, the CAC cohort was dominated by the group specific variant KRAS G12D, which accounted for 45.5% of all *KRAS* mutations in this group. This might support the suitability of this variant as a genetic marker for our purpose. In total, 44.2% of all CRCs show mutations in *KRAS*, of which 13.2% account for the KRAS variant G12D [26].

In IBD, the gene with the most prevalent mutation rate is *TP53*, where most of the variants affect the DNA binding domain that spans from codons 102–292 [27]. In our cohort, in approximately half of all cancer samples a *TP53* mutation was detected. With a proportion of 72.7% and 93.3% of CACs and sCRCs, the majority of these variants relate to the DNA binding domain. Additionally, it was reported that in CACs the commonly mutated codons 175, 245, 248 and 273 were rarely affected and more uncommon codons like 158, 179 and 342 were found with mutations [22]. Our study only confirms a small part of these findings. In total, two CAC samples showed an uncommon variant at codon 179, whereas none of the sCRCs were affected. Interestingly, with 26.7% the most common variant in the sCRC group affected codon 282, but the same variant was also detected in one CAC sample.

CACs often present special morphologies, known as the non-conventional types. Therefore, we re-evaluated our mutational datasets regarding conventional and the non-conventional type of CACs. Our data revealed that even if both types of carcinomas show comparable levels of mutations (90.5% and 88.2%), the affected genes differ from each other. Interestingly, ATM mutations only appeared in conventional CACs. So far, there is a lack of data that focusses on genetic differences of both CAC types. One study demonstrated that there are differences in LGR5 gene expression concerning CAC with conventional and non-conventional morphology [28]. Other groups also detected different molecular profiles for both types and described the morphology as quite heterogeneous [29, 30]. In addition, our samples of the conventional type were about 3.6 times more likely mutated in *kras* compared to the non-conventional type. This suggests that there are genetic differences between both cancer types, which might lead to the recommendation to treat them as different groups.

For CRC development, the microbiological environment in the colon also plays a role. Therefore, we correlated the mutational status with the presence of *F. nucleatum*, as both studies were performed on mostly the same patient cohort. In general, our data indicates no relationship between the presence of *F. nucleatum* and the gene mutation rates in our cohort, as samples that were not affected by this bacterium showed similar mutation profiles. In 2019, a “two-hit” model was proposed [31] that extends the adenoma-carcinoma-sequence model [32]. It is proposed that driver mutations in the host represent the first step, followed by accumulation of microbes like *F. nucleatum*, which exacerbates cancer progression [31]. Also, another study concluded that an increasing burden of *F. nucleatum* during or after the transition of an adenoma to a carcinoma promotes the sessile serrated pathway (SSP), but not the KRAS-associated CRC carcinogenesis [33]. Their data showed, that 56% of CRCs with BRAF mutations were positive for *F. nucleatum*, whereas this was the case in only 44% of *F. nucleatum* negative cases. 44.6% of samples with *F. nucleatum* infection were mutated in KRAS at codons G12/ G13, while 55.4% of patients without *F. nucleatum* were mutated in this gene [33]. Our data is not consistent with these findings, as 28.6% (8/28) of *F. nucleatum* positive CRCs show mutations in BRAF, while this was the case for only 6.1% of negative CRCs (2/33). Also, we detected lower levels of KRAS mutations, in CRCs with and without *F. nucleatum* infection. By taking a look at CAC and sCRC data together, 32.1% (9/28) of CRCs with infection and 39.4% (13/33) without infection of this bacterium were mutated.

## Conclusion

We demonstrated that there are genetic differences between CACs and sCRCs, but also within the CAC group. The distinction according to conventional and non-conventional CAC revealed that especially ATM shows distinct mutation patterns between these groups. Because these morphological differences can already be observed in the pre-cancerous lesions, like LGD, further studies should also focus on these groups. So far, also the contribution of *F. nucleatum* to the progression or as a trigger for CRC development remains unsolved. While our data presented no correlation between the bacterium and CRC, it may still have a pathogenic effect on genes that were not included in this study. An extension of analyzed genes could give interesting insights on the connection between *F. nucleatum* and CRCs.

## Electronic supplementary material

Below is the link to the electronic supplementary material.

Supplementary Material 1

## Data Availability

No datasets were generated or analysed during the current study.

## Abbreviations

APC: Adenomatous Polyposis Coli
ATM: Ataxia Telangiectasia Mutated
BRAF: Raf Murine Sarcoma Viral Oncogene Homolog B1
CAC: colitis-associated carcinoma
CD: Crohn’s disease
CDKN2A: Cyclin Dependent Kinase Inhibitor 2 A
CRC: colorectal carcinoma
F. nucleatum: Fusobacterium nucleatum
FBXW7: F-Box and WD Repeat Domain Containing 7
FFPE: Formalin-fixed paraffin-embedded
GNAS: Guanine Nucleotide Binding Protein, Alpha
HE: Hematoxylin-eosin
HGD: High-grade dysplasia
IBD: Inflammatory bowel disease
KRAS: Kirsten Rat Sarcoma Viral Oncogene Homolog
LGD: Low-grade dysplasia
LGR5: Leucine Rich Repeat Containing G Protein-Coupled Receptor 5
MCR: Mutation cluster region
MET: Mesenchymal Epithelial Transition
NGS: Next-generation sequencing
PDGFRA: Platelet Derived Growth Factor Receptor Alpha
PIK3CA: Phosphatidylinositol-4,5-Bisphosphate 3-Kinase Catalytic Subunit Alpha
PTEN: Phosphatase And Tensin Homolog
qPCR: Quantitative real-time polymerase chain reaction
sCRC: Sporadic colorectal carcinoma
SMAD4: Mothers Against DPP Homolog 4
SNP: Single-nucleotide polymorphism
SSP: Sessile serated pathway
UC: Ulcerative colitis
TP53: Tumor Protein 53
