# Supplementary material for: Mutational analysis differentiating sporadic carcinomas from colitis-associated colorectal carcinomas
Source: Cell Commun Signal. 2024 Oct 10;22:483. doi: 10.1186/s12964-024-01856-8 (PMC11465924; doi:10.1186/s12964-024-01856-8)
Supplement: Supplementary file 1 — Supplementary Material 1 [file 12964_2024_1856_MOESM1_ESM.docx]

Supplementary Figure

**Mutational analysis differentiating sporadic carcinomas from colitis-associated colorectal carcinomas**

Theresa Dregelies^1,2^, Franziska Haumaier^1^, William Sterlacci^1^, Steffen Backert^2^, Michael Vieth^1,3^

^1^Institut für Pathologie, Friedrich-Alexander-Universität Erlangen-Nürnberg, Klinikum Bayreuth, Preuschwitzer Str. 101, 95445 Bayreuth, Germany

^2^Lehrstuhl für Mikrobiologie, Friedrich-Alexander-Universität Erlangen-Nürnberg, Staudtstr. 5, 91058 Erlangen, Germany

^3^Bavarian Cancer Research Center (BZKF), Erlangen, Germany

^*^Corresponding author: Prof. Dr. med Michael Vieth, E-Mail: michael.vieth@fau.de, ORCID: 0000-0002-4336-7721, Tel.: +49 921/400-5602, Fax: +49 921/885602


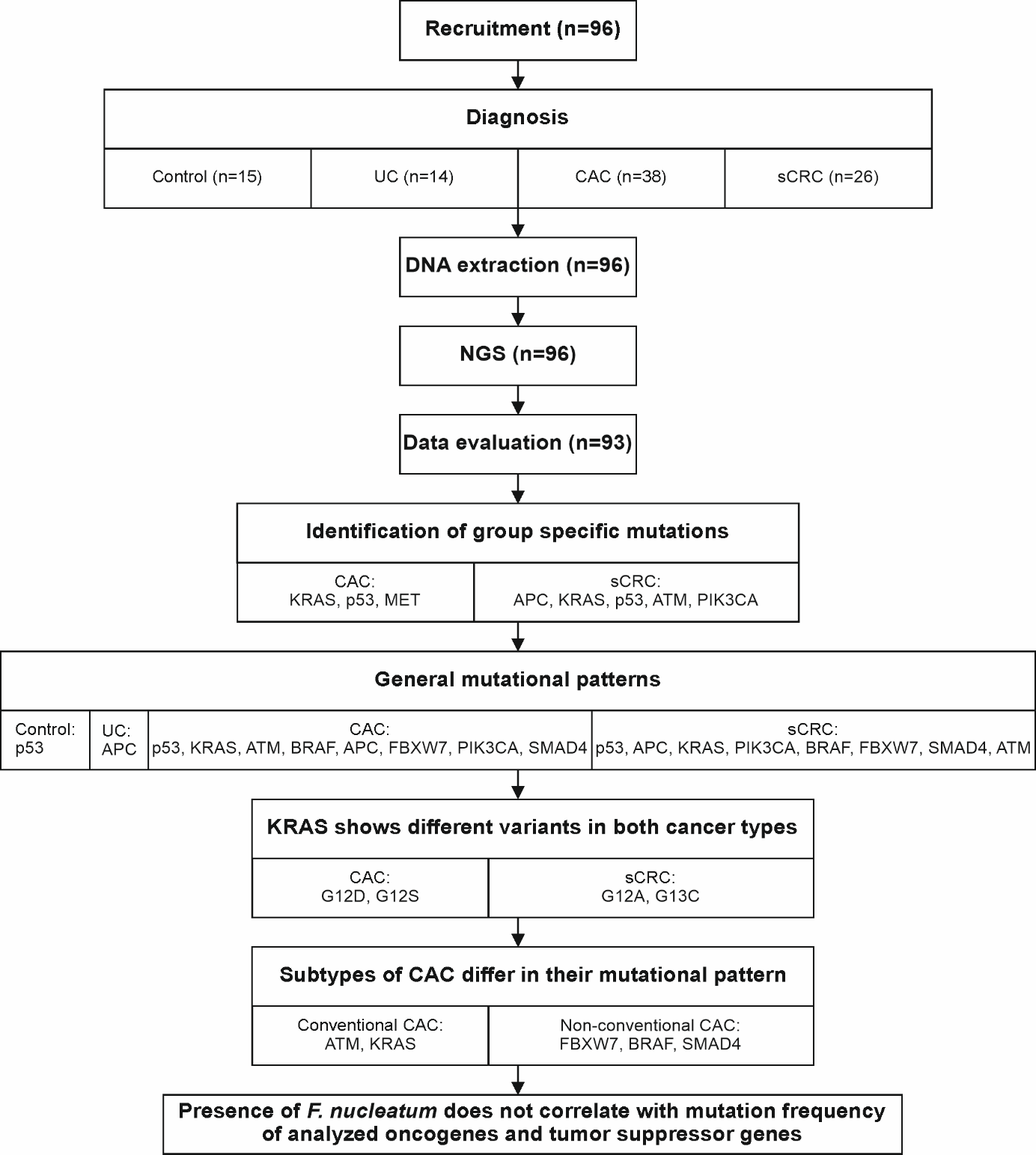


Fig. S1: Flowchart of the study design.
